# P01-045 – Epilepsy in Armenian children with FMF

**DOI:** 10.1186/1546-0096-11-S1-A48

**Published:** 2013-11-08

**Authors:** N Mkrtchyan, G Amaryan, N Aghababyan, K Mirzabekyan, T Sarkissian

**Affiliations:** 1 National Pediatric Centre for Familial Mediterranean Fever of Arabkir Joint Medical Centre - Institute of Child and Adolescent Health, Armenia; 2 National Pediatric Centre for Familial Mediterranean Fever of Arabkir Joint Medical Centre - Institute of Child and Adolescent Health, Yerevan State Medical University, Armenia; 3 Arabkir Joint Medical Centre - Insitute of Child and Adolescent Health, Yerevan, Armenia; 4 Arabkir Joint Medical Centre - Institute of Child and Adolescent Health, Yerevan, Armenia; 5 Centre of Medical Genetics and Primary Health Care, Yerevan, Armenia

## Introduction

FMF is an ethnically restricted disease for ancestors from Mediterranean sea region and it has high prevalence in Armenia as well. Neurologic manifestations in children with FMF are relatively uncommon. Headaches occur frequently in FMF and have mainly migraine-like nature. Aseptic meningitis and convulsive disorders as well have been reported.

## Objectives

To study the epilepsy in Armenian children with FMF.

## Methods

We observed 2300 patients with FMF (1408 boys and 892 girls; mean age: 8.86±0.29) in the National Pediatric Centre for FMF. Diagnosis of FMF was based on Tel-Hashomer criteria and MEFV genetic analysis. The epilepsy was diagnosed based on clinical manifestations (>2 unprovoked epileptic seizures),neurological history, exam, EEG and MRI. The statistical analysis was performed using Epi-Info 2000 software.

## Results

Epilepsy was diagnosed in 12 (0.5%) FMF patients (5 boys and 7 girls; aged from 7 to 18 years). The frequency of the epilepsy was not exceed the average indices for a healthy Armenian population (0.6%). The mean age of FMF manifestation was 3.5 years and the same indices for epilepsy onset made 7.5 years. Family history on FMF and epilepsy was observed in 9 and 6 patients respectively.

FMF with moderate activity was diagnosed in most (8) patients. Four patients with severe course of FMF had acute recurrent arthritis. At that the following mutations of *MEFV* were detected: 694V/M694V (4 patients); M694V/V726A (3 patients) and by each one of M694V/R764H, M694V/E148Q, V726A/M680I, M694V/0, E148Q/0 (in total - 5 patients).High penetrance M694V mutation was determined in 5 patients, mainly with severe homozygous genotype (4 patients). Colchicine therapy was effective for 7 patients. Partial epilepsy with secondary generalized and/or complex partial seizures was diagnosed in 6 FMF patients. Four had primary generalized epilepsy with frequent polymorphic convulsions. Symptomatic epilepsy with polymorphic partial fits had two FMF patients with cerebral palsy. Idiopathic generalized epilepsy with photosensitive absences was in one case. In 7 FMF children epilepsy was diagnosed after manifestation of FMF and they were responsive to antiepileptic treatment. In 5 patients convulsive disorder precede the diagnosis of FMF. In these cases, convulsions were resistant to antiepileptic drugs alone and they subsided only when colchicine was added.

## Conclusion

Taking into account, that epilepsy is a genetically determinate disorder and the majority of FMF patients with epilepsy were carriers of M694V mutation and severe homozygous genotype, it is not excluded, that the combination of FMF with certain type of convulsive disorders/epilepsy (probably based on some vascular lesions) might be possible in the presence of similar environmental and social triggers. However, we suppose that the low frequency of epilepsy (0.5%) among Armenian children with FMF, which does not exceed the frequency for general healthy population, as well as the positive response of these patients to both colchicine and antiepileptic drugs indicate the presence of two separate diseases.

## Disclosure of interest

None declared.

